# Blackland Conservation and Utilization, Carbon Storage and Ecological Risk in Green Space: A Case Study from Heilongjiang Province in China

**DOI:** 10.3390/ijerph20043154

**Published:** 2023-02-10

**Authors:** Chen Qu, Wen Li, Jia Xu, Song Shi

**Affiliations:** College of Landscape Architecture, Northeast Forestry University, Harbin 150000, China

**Keywords:** carbon storage, ecological risk, land use policy, green space, carbon neutrality

## Abstract

Clarifying the relationship between carbon storage and ecological risks is critical to ensuring regional sustainable development. Land use changes caused by land use policy invariably result in substantial changes in carbon storage and ecological risks. The link between carbon storage and ecological risks in green space is still unknown, even though green space is an essential ecological function carrier. According to the Blackland Conservation Utilization (BCU) policy document and natural exploitation (NP) status, this study compared and projected the carbon storage and landscape ecological risk characteristics of green space in Heilongjiang Province (HLJP) for 2030. It also quantitatively assessed the interactions and synergistic changes of the two variables in terms of coupled coordination relationships, quantitative correlations, and spatial correlations. The results demonstrated the following: (1) the green space evolution of HJLP under the BCU scenario is significantly more drastic than under the NP scenario; (2) In 2020–2030, the NP scenario’s evolution of green space results in the ecosystem losing 323.51 × 10^6^ t of carbon storage, compared to the BCU scenario’s loss of just 216.07 × 10^6^ t. The BCU policy will increase the agglomeration of high-risk ranges in the northeast and southwest will but decrease the overall landscape ecological risk level of green space; (3) BCU policy will prevent the system’s orderly development and benign coupling, but it will increase the interdependence between carbon storage and landscape ecological risks in green space; (4) Green space exchange and loss will result in the simultaneous rise or decrease in both variables. The magnitude of carbon storage increase owing to green space expansion tends to increase simultaneously with the magnitude of landscape ecological risk reduction. To a certain extent, the HLJP black land conservation and utilization policy can improve carbon storage and ensure ecological security, and the matching of dominant regions with the status of the landscape evolutionary process can support future carbon-neutral actions.

## 1. Introduction

The greatest ecological risk and environmental crisis in recorded history are being brought on by rising carbon dioxide concentrations, which are also causing extreme disaster phenomena [1,2]. Land use and cover change (LUCC) influences carbon cycling by altering the spatial and temporal patterns of ecosystems, and governments have all decided to increase terrestrial ecosystem carbon stocks to offset CO_2_ emissions from societal development [3,4]. The implementation of the action plan to address climate change has been on the agenda since China proposed, in 2020, to strive to achieve the carbon peak by 2030 and strive to complete the carbon-neutral target by 2060 [5]. Agriculture and forestry are two policy-oriented engineering techniques that have been found to increase ecosystem carbon storage. These practices also have an impact on how biological processes respond, which changes the level of risk control in regional landscape patterns [6,7]. To inform LUCC and spatial planning decisions under the carbon neutrality target direction, it is still unknown how the capacity of ecosystems to store carbon and landscape patterns are related. Therefore, more research is required to comprehend the connection between landscape security patterns and carbon sequestration functions.

Carbon storage (CS) is a climate-regulating ecosystem service (ES) function, and LUCC is a crucial determinant of carbon storage in terrestrial ecosystems [8,9]. Evaluations of landscape ecological risk (LER) can show how much the landscape mosaic deviates from the ideal pattern and can identify potential risks or pressures brought on by ecosystem structure and function [10,11]. However, considering carbon storage alone cannot describe the spatial pattern of the landscape, and considering ecological risk alone cannot reflect the processes and functions of ecosystems, even though studies on CS and LER as two distinct scientific questions have been conducted more generally [12,13]. Studies relating CS to LER are progressively enriched as well. On the one hand, the assessment of landscape ecological risk is based on ecosystem services, such as the function of carbon sequestration as an indication of ecosystem service qualities or disclosing material–energy cycling processes, such as carbon and water balance [14,15]. However, the mechanisms tying ecosystem services and ecological risks together are unclear, because this approach focuses on identifying pertinent ecological risks. On the other hand, the relationship between ES and LER is investigated, including the application of the coupling coordination model [16], the gray correlation analysis model [17], and spatial correlation [18,19,20], in order to evaluate the correlation and level of interaction between the two. For instance, Gong et al. regard CS measurement and LER evaluation to be equally important study areas and offer a fresh viewpoint to merge the two [21]. The study demonstrates that there is a spatial relationship between carbon storage and landscape ecological risk levels in the western Chinese mountainous Bailong River basin, and that the reduction in ecological risk brought on by changes in land use is directly reflected in an increase in carbon storage services. In general, studies on CS and LER correlations are being progressively enriched; however, the majority of these studies are concentrated on evaluating CS and LER correlations at fixed moments and fixed areas, without taking into account how the evolution of the landscape pattern may affect the relationship between variables. This one-sided cognition prevents us from fully describing the intrinsic correlation properties of ecological processes and landscape structure. However, future scenario predictions are limited and rarely capture probable trends and outcomes for ecosystems [16].

Green space is the focus of the territorial spatial green space system planning, which also acts as a vital carrier for the function of carbon sequestration. In China, the territorial spatial planning system is a key tool for achieving the development of an ecological civilization. Compared to gray spaces, green spaces are more successful in performing ecological services [22]. These green spaces typically consist of forests, grasslands, waters, wetlands, etc., with forests and grasslands playing a crucial role in carbon sequestration and storage [23,24,25,26]. Heilongjiang province (HLJP) is located in the black soil region of Northeast China, which is the third largest black soil region in the world in terms of area, and the province has a large number of green space resources. However, during the past 70 years, the extent of black land reclamation has grown, and the ecosystem has evolved such that man-made agriculture has taken the place of natural grasslands and forests [27]. According to “The Regulations on the Protection and Utilization of Black Land in Heilongjiang Province,” locations with black soil will be subject to rigorous use controls. How will the ecological risk vary while modifying the carbon storage of green space, and what will be the connection between them? These questions will be influenced by the black land conservation and usage policy. This study includes the following innovations: (i) concentrating on the study subject of CS and LER correlation to green space, which enhances the theoretical framework of the study of green space; (ii) incorporating the pattern evolution process of green space into the research of the correlation between CS and LER expands the range of related research with a novel viewpoint; (iii) attempting to integrate green space with black land conservation and utilization policy, which can improve the interface between land use policy and green space system planning; (iv) evaluating the potential ecological effects that a land use policy may have, which provides data for a policy benefit analysis.

Overall, this study takes HLJP as an example; the InVEST model and CA–Markov model were combined to quantitatively simulate the impact of black land conservation use on the carbon stock of green space, and the model was constructed to realize the measurement of landscape ecological risk. The objectives of this study are (1) to determine the potential effects on CS and LER in the green space of black land conservation and utilization policies; (2) to analyze the CS and LER’s coupled and coordinated interactions in green space; and (3) to use green space as a medium for the analysis of the links between CS changes and LER changes during the evolution of landscape patterns. As a theoretical foundation for regional territorial spatial planning and land policy formulation, the research findings are promising.

## 2. Materials and Methods

### 2.1. Study Area and Data

**Figure 1:**
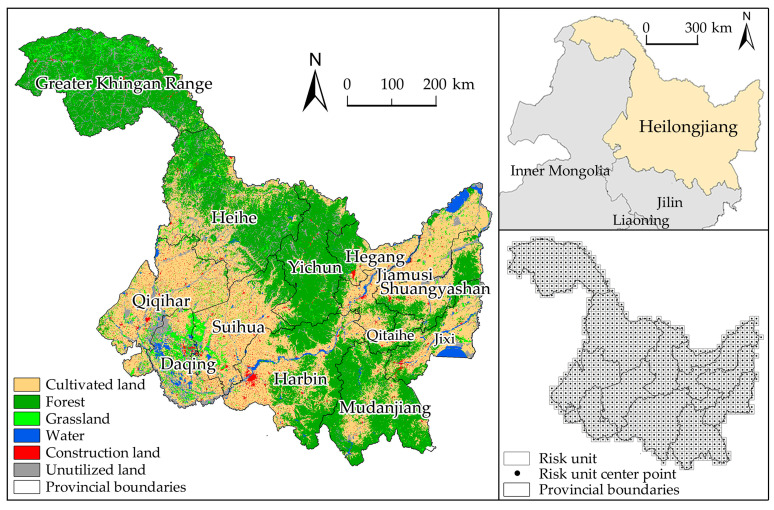
Geographical location, municipal administrative boundaries, and ecological risk unit division in Heilongjiang province. Land use data in the graphic are for the year 2020.

HLJP is the northernmost and easternmost provincial administrative region in China (43°26′ N~53°33′ N, 121°11′ E~135°05′ E), with 12 prefecture-level cities and the Great Khingan Mountains region, covering a total area of 473,000 km^2^ (Figure 1). HLJP has a cold-temperate continental monsoon climate with an average annual temperature between −5 °C and 5 °C, precipitation between 400 and 650 mm, and 83% to 94% of the total annual precipitation falling during the growing season. The vegetation accumulates a great deal of organic matter that is not suitable for decomposition, and the unique conditions form the black soil humus layer [27]. HLJP Black soil resources are extensively spread over cultivated land, forest land, grassland, wetland, and lake ecosystems, and black soil has a high organic matter content and a tremendous carbon sequestration capacity [28,29]. According to the White Paper on Northeast Black lands (2020) of the Chinese Academy of Sciences, black soils are at a high risk of degradation, including from frequent soil erosion on sloping arable land between 3 and 15 degrees and a progressive loss in ecosystem carbon reserves. Regional risks are exacerbated by factors including strong winds, tillage techniques, snowmelt runoff, and freeze–thaw cycles [30,31,32]. In 2021, the HLJP government and the Development and Reform Commission proposed the Regulations on the Protection and Utilization of Blackland in Heilongjiang Province and the 14th Five-Year Plan, respectively, to promote the conservation and intensive utilization of black land, construct a new pattern of spatial development and national protection, and enhance the service function and stability of the ecosystem.

Land-sat-TM/ETM images from 2000 and 2010 and Landsat 8 images from 2020 were interpreted to produce land use maps with a spatial resolution of 1 km that met the accuracy requirements of the study. According to the China Land Use Status Classification Standard (GB/T21010-2017), land use is divided into (1) cultivated land, (2) forests, (3) grasslands, (4) waters, (5) construction land, and (6) unutilized land. The Digital Elevation Model (DEM) data with a resolution of 90 × 90 m were obtained using the most recent SRTM V4.1 data by resampling, and the slope data were derived using the DEM data. The Chinese Academy of Sciences (http://www.resdc.cn accessed on 6 December 2022) was consulted for spatially interpolated annual mean temperature and precipitation, DEM data, and land use data. Black soil distribution, national roads, provincial roads and highways vector data were obtained from the National Earth System Science Data Center (http://www.geodata.cn accessed on 6 December 2022). The Heilongjiang Statistical Yearbook (http://tjj.hlj.gov.cn accessed on 6 December 2022) was consulted for its population and GDP figures. Additionally, all data were projected to the Krasovsky_1940_Albers coordinate system to maintain spatial consistency during data processing.

### 2.2. Simulation and Forecasting of Land Use Based on the CA–Markov Model

China picked the year 2030 as a pivotal point to achieve its carbon-peaking goal and connect carbon-neutral routes [33]. Due to the time-sensitive aspect of land use policy and spatial planning, the year 2030 was chosen as the time node for the LULC pattern projection. In this study, the CA–Markov model is utilized within the IDRISI17.0 program to implement scenario setting and LULC diagram simulation of HLJP in 2030. The following are the specific steps.

**Figure 2:**
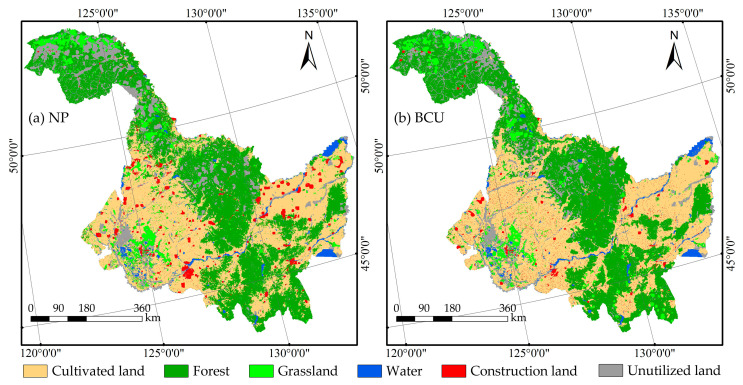
Land use simulations for 2030 under two scenarios. Notes: (**a**) NP scenario; (**b**) BCU scenario.

(1) Calculate the probability and area matrices of transfer. Based on the land use maps for 2000 and 2020, the transfer probability matrix and the transfer area matrix were derived. (2) Obtain the maps of land use change adaptability. To more accurately mimic the land use map of Heilongjiang Province, the MCE model was utilized to create the land use change suitability atlas. The analysis considers land use change scenarios based on two natural development (NP) and black land conservation use states (BCU). Considering previous research results [34,35], four natural drivers, including elevation, slope, temperature, and precipitation, and five social drivers, including population density, average local gross domestic product, distance from national roads, distance from provincial roads, and distance from highways, were selected, and limiting factors were inputted to generate an atlas of suitability rules for two scenarios. The NP scenario estimates the land use pattern in 2030 based on the trajectory of land use change in Heilongjiang Province between 2000 and 2020; The BCU scenario is based on the control requirements in the Regulations on the Protection and Utilization of Blackland in Heilongjiang Province, which (i) prohibit the conversion of land type into building land and arable land into other land types, state that (ii) cultivated land with a slope greater than 15 degrees cannot be developed, that (iii) cultivated land with a slope greater than 15 degrees will be turned into forest land and grassland and cannot be converted into other land types, and that (iv) the conversion of construction land to occupy black land is strictly controlled. (3) Check the simulation and correctness of a land use map. Using the 2010 LULC, transfer probability matrix and area matrix, and suitability rule atlas, a 5 × 5 CA filter with 10 iterations was utilized to simulate the 2020 LULC under two scenarios [36]. The kappa coefficient was utilized to determine the degree of consistency between the simulated image results of the land use pattern and the actual image data. The kappa coefficients for the NP and BCU scenarios are 0.85 and 0.79, respectively, which are both greater than 0.75 and satisfy the simulation requirements. (4) Predicting future land use patterns. The CA–Markov model was used to verify rules to simulate the LULC in 2030 under the ND and BCU scenarios (Figure 2).

### 2.3. Green Space Evolution Defined

Combining the definition of green space by research academics with the Chinese land resource classification system, green space was classified into four types: forest land, cultivated land, water and grassland, while construction land and unused land were labeled as non-green space [26,37]. According to the purpose of the study and concerning the research method of Chen et al. [38], green space exchange denotes the interconversion between different types of green space, green space loss denotes the transformation of green space to non-green space, and green space expansion denotes the transformation of non-green space to green space. These three types were used to categorize the different evolutionary types of green space.

In the manner previously described, the LULC image of 2020 was superimposed with the LULC image of 2030 obtained from the prediction simulation for analysis, and if the images of the two periods before and after were changed from forest land to construction land, it was described as green space loss; if they were, respectively, forested land and grassland, it was indicated as green space exchange. The map of the dynamic evolution of green space for 2020–2030 was then produced.

### 2.4. Carbon Storage Assessment Based on InVEST Model

The study utilized the Carbon Storage and Sequestration module of the InVEST 3.9.0 model, which is widely utilized in regional carbon stock simulation and assessment [39,40]; it employs land use types as assessment units, and based on four carbon pools, can directly quantify the influence of land use change on regional carbon storage. The modeling and evaluation of regional carbon storage data rely heavily on this model [41]. The formula for calculating carbon storage is as follows [42]:

(1) $$C_{i}=C_{i,above}+C_{i,below}+C_{i,soil}+C_{i,dead}$$

(2) $$C_{total}=\sum_{i=1}^{n}C_{i}\times S_{i}$$

where i is the land use type, $C_{i}$ is the total carbon density of land use type i (t/ha), $C_{i,\mathrm{above}}$ is the carbon density of above-ground part of the vegetation of land use type i (t/ha), $C_{i,below}$ is the carbon density of below-ground part of the vegetation of land use type i (t/ha), $C_{i,soil}$ is the soil organic carbon density of land use type i (t/ha), $C_{i,dead}$ is the carbon density of dead organic matter of land use type i (t/ha), *C_total* is the total carbon stock, and $S_{i}$ is the area of land use type i. Carbon density data were obtained based on previous research results [43,44,45,46,47], and field measurements of HLJP were preferred; if data comprehensiveness was insufficient, measured or data compiled from the literature near HLJP and in the same climate zone were used (Table 1).

To create the HLJP green space CS maps for the two scenarios in 2020 and 2030, the regional CS data from the same period were extracted using the green space distribution data mask. To produce the CS change maps of the various evolution types of green space for HLJP in the ND and BCU scenarios from 2020 to 2030, the regional CS change maps and green space evolution type maps were combined.

### 2.5. Landscape Ecological Risk Assessment Model

Concerning earlier research techniques, this study built the HLJP ecological risk evaluation model based on the characteristics of the landscape pattern [48,49,50,51,52,53,54] (Table 2). By the principle of 2–5 times the average area of landscape patches in the study region [55], the study area was divided at equal intervals into a 20 km × 20 km rectangular grid, and 1273 ecological risk plots were obtained. The ERI was assigned to the center of the respective risk zone, and the LER spatial distribution of HLJP was determined using the Kriging interpolation technique. The LER raster data were classified into five rank bands of low risk (<0.010), lower risk (0.010–0.012), medium risk (0.012–0.013), higher risk (0.013–0.015) and high risk (>0.015) using the natural breakpoint method.

Green space distribution data from the same period were masked with regional LER data to produce an LER map of green space for the same period. The raster calculator tool in ArcGIS10.8 was used to obtain regional LER change data for two scenarios from 2020 to 2030, which were then extracted using the green space evolution type map mask to provide LER change maps of various evolution types in green space.

### 2.6. Coupling Coordination Degree Model

The coupling coordination degree was employed to analyze the coupling degree and coordination relationship between the two variables based on the construction of the coupled correlation function between carbon storage and the landscape ecological risk of HLJP green space. The formula is as follows [57]:

(3) $$C=2{\left\{\left(W\times S\right)∕{\left(W+S\right)}^{2}\right\}}^{\frac{1}{2}}$$

(4) T=α⋅W+β⋅S

(5) $$D={\left(C\times T\right)}^{\frac{1}{2}}$$

In the formula, W is the carbon storage of green space, S is the landscape ecological risk of green space, C represents the coupling degree of carbon storage and landscape ecological risk of green space, T represents the evaluation index of coupled and coordinated development, α and β are both coefficients to be determined, and the study considers CS and LER as equally important research categories [21]; α and β are assigned a value of 0.5, and *D* is the coupling coordination degree. To exclude the influence of *D* and S on the dimensional, the data were normalized to the interval [0, 1] using the polar difference standardization method [16].

### 2.7. Bivariate Spatial Autocorrelation Model

The study utilized a bivariate spatial autocorrelation model and GeoDA 1.14.0 software to explore the degree of spatial location-based correlation between CS and LER changes in the green space of HLJP.

The bivariate Global Moran’s index uses a formula to determine the degree of spatial aggregation between two variables [58]:

(6) $$I=\frac{n\Sigma_{i}^{n}\Sigma_{j}^{n}W_{ij}Q_{k}^{i}Q_{l}^{j}}{\left(n-1\right)\Sigma_{i}^{n}\Sigma_{j}^{n}W_{ij}}$$

The bivariate Local Moran’s index represents the degree of association and difference between the two variables locally in space, and is calculated as follows [57]:

(7) $$I_{s}=Q_{k}^{i}\Sigma_{j=1}^{n}W_{ij}Q_{l}^{j}$$

where $Q_{k}^{j}=\left(X_{k}^{i}-{\bar{X}}_{k}\right)/\delta_{k}$, $Q_{l}^{j}=(X_{l}^{j}-{\bar{X}}_{l})/\delta_{l}$; n is the number of spatial units (divided into 1273 evaluation units in this study), $X_{k}^{i}$ is the value of attribute k of spatial unit i; $X_{l}^{j}$ is the value of attribute l of spatial unit j; ${\bar{X}}_{k}$,$\;{\bar{X}}_{l}$ are the mean of attribute k, l; $\delta_{k}$, $\delta_{l}$ are the variance of attribute k, l; $W_{ij}$ is the weight matrix measuring the adjacency relationship between spatial units [59]. I takes values in the range of [−1, 1], and less than 0 indicates a negative spatial correlation, equal to 0 indicates no correlation, and greater than 0 indicates a positive correlation [60]. The local spatial autocorrelation analysis was performed using the local indicator of spatial association (LISA) with the change value of CS as the first variable and the change value of LER as the second variable.

## 3. Results

### 3.1. Potential Impact of Black Land Conservation and Utilization Policies on the Dynamics of Green Space

**Figure 3:**
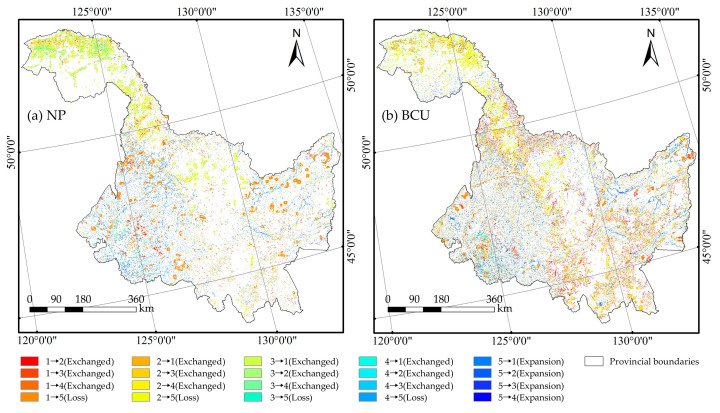
The dynamic evolution of green space from 2020 to 2030. notes: 1–5 are Cultivated land, forest, grassland, water and non-green space respectively. (**a**) NP; (**b**) BCU.

During 2020–2030, under the BCU scenario, the evolution of green space in HLJP is significantly more dramatic than under the NP scenario (Figure 3). The most important expansion of green space during the next ten years will be the expansion of cultivated land, while the conversion of some non-green areas to forests and grasslands will also be substantial, particularly in the Great Khingan in the northwest and the Songnen Plain in the southwest. In the BCU scenario, the extent of forest conversion to cultivated land and grassland decreases more than in the NP scenario, while the area of grassland conversion to cultivated land and forest increases significantly, indicating an overall expansion of cultivated land and a gradual loss of forest and grassland in the exchange of categories across green space. Of note is that the conversion of cultivated land to forest and grassland occurs primarily in the northeast and southwest of HLJP; based on this, the above conversion occurs significantly along the mountain’s edge under the BCU scenario, characterizing the significant results that will be achieved by the policy of returning cultivated land to forest and grassland on sloping land. Under the BCU scenario, the conversion form of cultivated land to non-green space shifts from blocky to scattered, and the degree of cultivated land loss is successfully reduced.

In both scenarios, the area of green space loss is significantly more than the area of green space expansion, indicating that the area of green space in HLJP will indicate a tendency of decline (Table 3). Compared to the NP scenario, the BCU scenario demonstrates a more significant increase in green space and a loss reduction. The BCU measures would prevent the loss of 8492 km^2^ of green area overall.

### 3.2. Potential Impacts of Blackland Conservation and Utilization Policies on Carbon Storage and Landscape Ecological Risks in Green Space

#### 3.2.1. Potential Impact on Carbon Storage in Green Space

**Figure 4:**
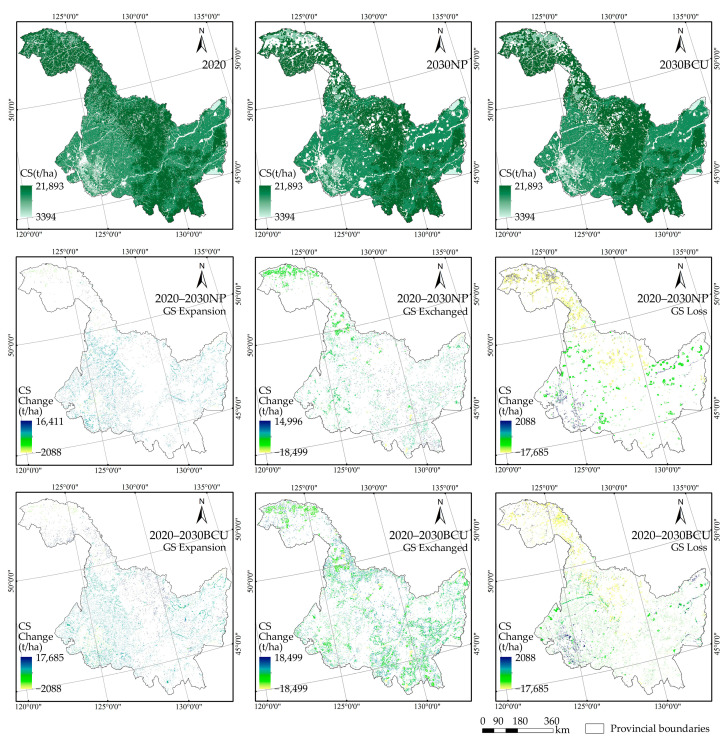
Spatial distribution of carbon storage in green space (GS) and its changes in two scenarios from 2020–2030.

The spatial distribution of carbon storage in the green space of HLJP in the 2030 NP scenario is high in the southeast, northwest, and central regions of the province and low in the northeast and southwest (Figure 4); the range in carbon storage in the green space within the large and Lesser Khingan Mountains (eastern Heihe City and western Hegang City) and the Sanjiang Plain (Jiamusi City) in the BCU scenario demonstrates a significant increase compared to the NP scenario. Compared to 2020, the change in carbon storage in the green area of HLJP under the BCU scenario in 2030 is much greater than under the NP scenario. Specifically, the growth of green space under the NP scenario causes the ecosystem to lose 323.51 × 10^6^ t of carbon stock, whereas, in the BCU scenario, the carbon loss is only 216.07 × 10^6^ t. In the BCU scenario, the evolution of green spatial exchange is accompanied by the greatest carbon storage change, and the territorial pattern of green spatial carbon storage change is in part dictated by this condition. Notably, the 2030 BCU scenario significantly mitigates the decline in carbon storage in the southern and northeastern portions of the HLJP under the NP scenario but exacerbates carbon loss in the northern portion of the Great Khingan. Overall, the BCU scenario will promote the expansion of cultivable land and inhibit the transfer of cultivable land to non-green space, such as construction land, which will result in a significant increase in the carbon storage fixed in the green space expansion state and a reduction in the carbon storage released in the green space loss state, thereby preventing the loss of eco-system carbon storage.

#### 3.2.2. Potential Impact on the Ecological Risk of the Landscape in Green Spaces

**Figure 5:**
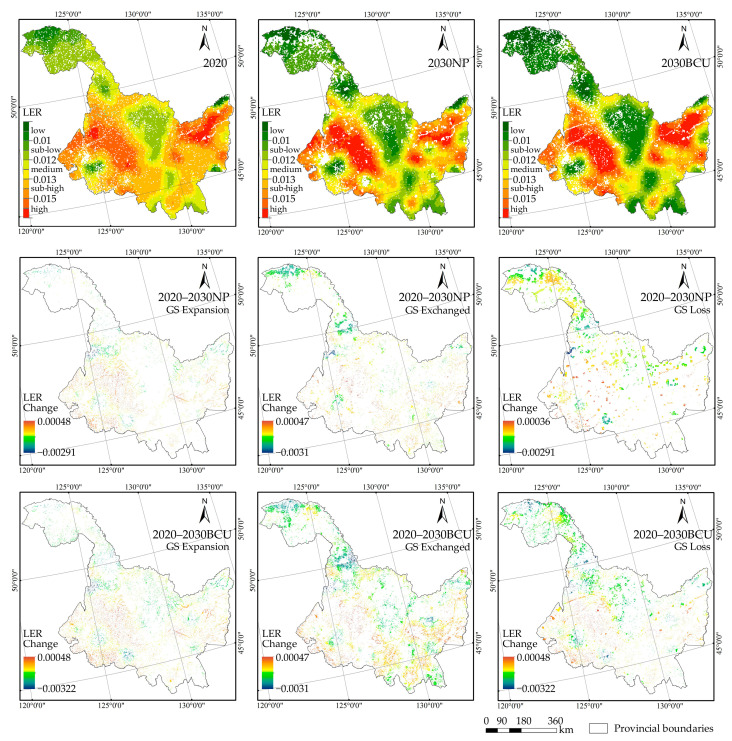
The spatial distribution of landscape ecological risks for green space (GS) and its changes under the two 2020–2030 scenarios.

The LER results for green space in 2020–2030 (Figure 5) show that the spatial pattern of landscape ecological risk in both scenarios is high in the northeast and southwest, and low in the southeast, northwest, and central. However, the extent of high-risk areas will tend to cluster more in the northeast and southwest of HLJP, intensifying the ecological risk of the region. In comparison to the NP scenario, the BCU scenario can significantly increase the area of low-risk areas, effectively reducing the overall landscape ecological risk level of green space. The difference in the geographical patterns of ecological risk changes brought on by the growth of green space between the two scenarios over the ensuing ten years is mostly attributable to the decrease in ecological risk in the western part of Harbin and Yichun City. In both scenarios, landscape ecological risk in the northern and southeastern portions of Greater Khingan was reduced, which was mostly attributable to the evolution of green space interchange, but the ecological risk reduction effect was more pronounced in the BCU scenario. Compared to the NP scenario, the degree of ecological risk change caused by the evolution of green space exchange in HLJP in the BCU scenario is more severe. Noticeably, the ecological risk in the southwestern part of Heihe City and the eastern part of Hegang City is effectively reduced in the NP scenario, and some of the high-risk areas are transformed into low and medium-risk areas, but this transformation does not occur in the BCU scenario. The BCU scenario effectively limits the non-green space connectivity to occupy the green space, and promotes the implementation of the sloping cultivation land reforestation and grass restoration project; this is also a significant reason for the transformation of higher risk level areas into medium and low-risk areas within the green space at the provincial scale.

### 3.3. Coupled Coordination Analysis of Carbon Storage and Landscape Ecological Risk in Green Space

In 2020–2030, the HLJP coupling coordination degree decreases from 0.865 to 0.838 (NP scenario) and 0.83 (BCU scenario), and the total coupling coordination degree increases (Table 4). Overall, HLJP has a lower CS and a higher LER level. The higher coupling degree of CS and LER in the BCU scenario compared to the NP scenario and the lower coupling coordination degree indicate that the BCU policy will increase the degree of interaction between the two variables but limit the system’s orderly development.

**Figure 6:**
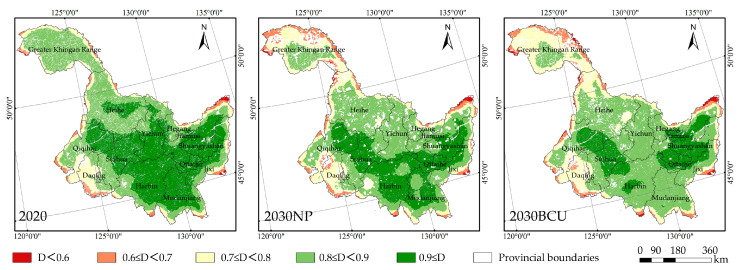
Spatial distribution of the coupled coordination of carbon storage and landscape ecological risk in green space.

The spatial pattern shows a high coupling coordination level in the southeast and a low coupling coordination level in the northwest in the NP scenario from 2020 to 2030; however, there is a significant reduction in the high coupling coordination (D > or equal to 0.9) area in the cities of Heihe, Yichun, and Jiamusi (Figure 6). Based on the NP scenario, the degree of coupling coordination within Harbin and Mudanjiang in the BCU scenario will be significantly reduced; meanwhile, the high coupling coordination area in Jiamusi will be diffused, and the diffused area will consist primarily of cultivated land; the degree of coupling coordination demonstrates a spatial pattern of high in the northeast and southwest, and low in the northwest and southeast.

### 3.4. Correlation Analysis of Carbon Storage Changes and Landscape Ecological Risk Changes in the Evolution of Green Space

#### 3.4.1. Quantitative Correlations

**Figure 7:**
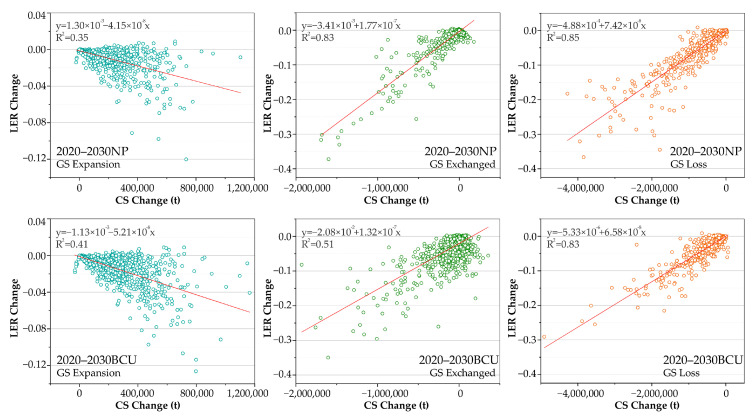
Scatter plot of carbon storage changes and landscape ecological risk changes of green space (GS) evolution per unit area in the study area.

Both CS and LER variation value scatter plots have a radial distribution pattern (Figure 7). Based on the SPSS K-S (Kolmogorov-Smirnov) normality test, neither the unit area CS change nor the LER change followed a normal distribution. Since it was not feasible to apply Pearson correlation analysis, Spearmen correlation analysis was employed to determine the correlation between the variables. The correlation coefficients for the green space expansion, exchange, and loss states in the 2020–2030 NP scenario are −0.712, 0.493, and 0.926, respectively, showing a strong correlation; this indicates that the green space expansion in this scenario leads to a larger increase in CS, while the decrease in LER tends to increase. Conversely, the green space exchange and loss will lead to a larger increase in CS, and the LER increase or decrease simultaneously become larger. The correlation coefficients of the three evolutionary stages of green space in the BCU scenario are −0.666, 0.340, and 0.814, which are all slightly lower than those in the NP scenario, indicating that the BCU policy will lessen the correlation between the CS changes and LER responses.

#### 3.4.2. Spatial Correlations

**Figure 8:**
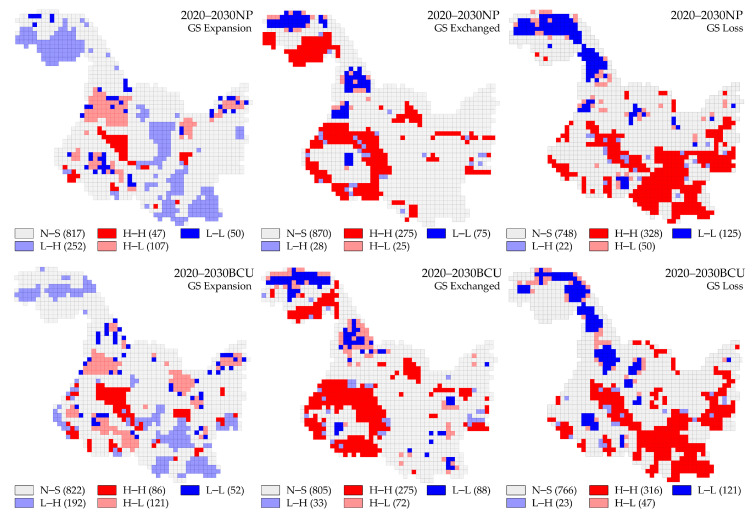
Spatial correlation of carbon storage changes and landscape ecological risk changes in the evolution of green space (GS) per unit area in the study area.

The global Moran’s indices (*p* = 0.05) for the evolutionary states of green space expansion, exchange, and loss over the next decade were −0.310, 0.414, and 0.312, respectively, in the NP scenario, and −0.254, 0.251, and 0.260, respectively, in the BCU scenario; this indicates that there is a negative spatial correlation between CS changes and LER changes triggered by green space expansion within HLJP and that there is a positive spatial correlation between the CS change and LER change, induced by green space exchange and loss. The green space exchange state in the NP scenario was most sensitive to the spatial relationship between CS change and LER change, but the influence of each evolutionary stage of green space on the variable relationship was not significantly different in the BCU scenario. The results of the local bivariate spatial autocorrelation LISA analysis (Figure 8) categorized the association patterns of CS change and LER change into five categories: high CS change–high LER change (H–H), low CS change–low LER change (L–L), low CS change–high LER change (L–H), high CS change–low LER change (H–L), and insignificant (N–S) patterns.

In the evolution of green spatial expansion, the H–H region in the NP scenario has the smallest proportion (3.69%) and is primarily concentrated in Suihua city; the L–L region is distributed in a dotted pattern in Heihe, Jiamusi, and Suihua city; the L–H region has the largest proportion (19.8%) and is primarily distributed in the Great Khingan region and Yichun, Harbin, and Jiamusi city; and the H–L region is distributed in Heihe, Jiamusi, Suihua and Harbin city. Compared to the NP scenario, the H–H region in the BCU scenario rose by 82.9%, and the increased area was primarily found in Suihua city. The L–L region relocated from the outside of the study area to the interior, the L–H region decreased significantly in Great Khingan and Yichun, while the H–L region decreased in Heihe and increased in Harbin and Jiamusi city.

In the green spatial exchange evolution, compared to the NP scenario, the total share of the H–H area in the BCU scenario remains unchanged at 21.6%. However, the Great Khingan region in the northwest shifts to Daqing, Suihua, and Qiqihar city, making the H–H ring area in the southwest more apparent, and the L–H area enclosed within the ring H–H area migrates to the boundary; Heihe City is an advantageous area for future green space exchange evolution for carbon sequestration advantages and risk management, as this sort of zone increases significantly there. Some H–L and L–L zone point-like ranges also appear in the eastern portion of HLJP.

In the evolution of green space loss, the quantitative changes of each type of zone in both scenarios are not significant; the increase in H–H areas is located within Harbin city, increasing the degree of connectivity between H–H areas in the southwest of HLJP and H–H areas in the north of Qiqihar and Suihua city; this is primarily caused by the expansion of non-green space. Based on the NP scenario, the BCU scenario predicts an expansion of the L–L zones at the intersection of Suihua, Daqing, and Heihe.

Overall, the BCU scenario would greatly increase the area of H–L regions, particularly in Qiqihar, Suihua, Daqing, and Heihe, which are predominantly located in low-elevation areas and are used to enhance agricultural share. The study’s findings indicate that BCU policy potentially plays a favorable effect in boosting CS and ensuring ecological security to a certain extent.

## 4. Discussion

### 4.1. Potential Impacts of Land Use Policies on Carbon Storage and Landscape Ecological Risks in Green Spaces and Their Causes

The decline in CS in the BCU scenario was substantially less than in the NP scenario, mostly due to the varied transfer rules of different land use categories within the black soil area in the two scenarios. Compared to the NP scenario, the BCU scenario indicates a considerable increase in CS in all places, except for the northern Great Khingan region, particularly in Heihe City, and the southeastern portion of the province; this is primarily because the transfer of cultivable land is prohibited in the aforementioned regions. Meanwhile, carbon density and the transformed area are the primary elements impacting CS alterations as a result of green space evolution [61,62]. Since the carbon density of cultivated land, forest land, and grassland is significantly greater than that of non-green space, the main form of contribution to the quantity of carbon sequestration caused by green space changes is the growth of green space. Due to the insignificant change in water areas under the BCU scenario, the carbon sequestration benefits of water areas are not immediately apparent. This section’s findings can serve as an empirical study of land use policy-induced carbon stock changes [5,63].

Variations in LER are attributable to alterations in land pattern change [64,65,66]. In HLJP, the LER level in the Great Khingan region is mitigated because the separation and fragmentation of forestland and grassland patches will be significantly reduced, whereas in BCU, the landscape patches are more connected and grassland is more widely distributed, resulting in a more significant LER reduction effect; the green space exchange transformation from cultivated land to forests mainly occurs in the LER reduction area in Yichun. Conversely, in the BCU scenario, the LER was significantly alleviated, primarily by avoiding the phenomenon of non-green space occupying cultivated land, and the holistic degree of forest land was subsequently increased, altering the regional fragmentation and dispersion index; this is consistent with the findings of Shen et al.’s study on returning cultivable land to the forest [67]. The LER within the cities of Heihe and Hegang is higher than the natural state of development, primarily because these areas will secure more cultivable areas in flat terrain areas, increasing the dominance of cultivated land while reducing the overall diversity of regional patches; this will increase the potential level of disturbance in the ecosystem at the hands of external influences [55].

### 4.2. Relationship between Carbon Storage and Landscape Ecological Risk in Green Space in the Future Period

The quantitative and spatial–temporal connections between ecosystem services and landscape ecological risks vary substantially between regions [16,17,18,19,20,21,68]. There is no lack of research on the relationship between the two variables at the provincial scale; for instance, ecosystem services and landscape ecological risk in Fujian Province exhibited a significant negative correlation, with the value of regulatory service functions bearing the greatest impact intensity [69,70]. In actuality, existing studies only reflect the potential of ecosystem service enhancement and landscape ecological risk management, despite the fact that spatial and temporal heterogeneity characteristics are considered; however, the influence of landscape pattern change processes is disregarded, making it difficult to determine the degree of synergistic changes in ecosystem services and ecological risks [71,72,73]. This study’s major important contribution is that we identify three processes in the dynamic evolution of green space and utilize them to illustrate the interconnected consequences of CS and LER changes. To the best of our knowledge, this is the first attempt to incorporate landscape pattern development processes into the study of the relationship between ecosystem services and ecological risks, and it has the potential to significantly increase the range and depth of related research.

In this study, the CS and LER of green space within HLJP showed an excellent coupling and coordination relationship (D > 0.8), but continued urbanization will result in a minor decrease in coupling synergy in the future, which is similar to the findings of Zhang et al. [16]. The BCU policy will encourage the execution of projects, such as black land preparation and resource exploitation, the efficiency of intensive land use will be enhanced, intensifying landscape disturbance, and the relationship between CS and LER will be strengthened. It is clear that the BCU policy focuses primarily on black soil cultivated land, and a large number of areas with a point increase in cultivated land appear spatial throughout the HLJP, which affects the stability of the original ecological structure of each area and impedes the orderly coupling and development of the system. Significant correlations were seen between changes in CS and LER as a result of various evolutionary states in green space, as well as substantial discrepancies. In the green space expansion state, for instance, the two variables exhibit a negative connection, but the positive correlation in the loss state is much bigger than that in the exchange state, a phenomenon that confirms the good ecological effects of green space. The Spearmen correlation coefficients of CS changes and LER changes have decreased in the BCU scenario compared to the NP scenario, and the distribution patterns of various spatial relationship clusters tend to be more dispersed, which is consistent with the findings of the coupled coordination study. The transition of land types is closely related to the degree of synergistic changes in CS and LER. For instance, in Qiqihar and Suihua in the southwestern portion of HLJP, where arable land types predominate and various types of green space evolution frequently occur, the carbon density and vulnerability index of arable land are at high levels, causing the CS in this region to change dramatically and the LER to change to a greater extent. The synergistic changes in CS and LER both have the same magnitude and considerably different degrees of change, confirming that ecosystem services and landscape ecological risks have both inclusive and antagonistic interrelation phenomena [74,75]. Important objectives for land transformation include enhancing ecosystem services and ensuring ecological security [76]. The ecological consequences realized following policy implementation were anticipated by the spatially associated partitioning of CS and LER changes, with the different evolutionary states of green space reflecting the partitioning differences. L–H areas, for example, are widely distributed in clusters in green space expansion evolution and are scattered in other evolutionary states; this type of area is typically the least favorable for land preparation and transformation due to low CS variability and high LER variability in areas with decreasing CS and increasing LER (e.g., northern Suihua City). On the contrary, H–H zones are contiguously distributed in green space loss evolution and less distributed in expansion evolution, and regional areas with elevated CS and reduced LER in this type of region (e.g., forest and grass areas in the heart of Yichun City) play the most important role in enhancing CS and ensuring ecological security.

### 4.3. Implications for Black Land Conservation and Utilization Plan and Green Space System Planning of Territorial Spatial in the Future Stage

HLJP has a large number of black soil resources, and is a major region for land use policy and comprehensive landscape renovation, and is influenced by numerous policies and plans [77,78]. In the framework of territorial spatial planning, for instance, the scope of green space system planning has increased from focusing primarily on urban built-up regions, to the entire area scale of “mountains, water, forests, farms, lakes, and grasses” [79]. Overall, the study focuses on the regional-scale green space mechanism in the future period. On the one hand it demonstrates, through prediction, the potential environmental impacts that may be caused by the BCU policy, and on the other hand, the results that can be used as a theoretical basis for the preparation of green space system planning, providing a link between multiple plans and policies with the goal of carbon neutrality. On the basis of the study’s findings, we propose the following improvements to land policy and green space planning: (1) The results of the study indicate that the green space expansion state achieves ecological benefits and recommend the implementation of four zoning control strategies. (2) The H–L zone should be notably maximized for the development of land carbon sequestration projects and the active protection of green places that perform carbon sequestration functions. In addition, according to the BCU policy, Heihe city in the H–L zone is a superior area for promoting CS and controlling LER in this state and can serve as a pilot area for the development of future action plans. In fact, locations such as the intersection of Qiqihar, Suihua, and Daqing cities in the Songnen Plain, or the gently sloping areas in the cities of Hegang and Qitaihe have more ecological potential in the future and require heightened focus. (3) The replacement of green space in the H–H area should be actively pushed. The northern portion of Qiqihar city and the central portion of Suihua city are the dominant areas, and engineering measures, such as land preparation, should be carried out according to the optimal pattern of ecological security based on meeting the total amount of permanent basic farmland requirements. (4) L–H spaces should be strictly regulated, land sprawl should be regulated, and priority should be given to the establishment of green spaces that occupy significant spatial structures [80]. To offset the impact of human activities on the ecosystem, a specific ecological buffer zone can be moderately preserved in the L–H region (e.g., the northern portion of Suihua City) by constructing country parks and nature reserves. (5) The reuse of empty space in the L–L area should be guided in order to increase the proportion of carbon sequestration space and the total carbon storage of green space. (6) Because of the spatial heterogeneity of ecosystems [10], some areas with significant CS and LER effects are located at the intersection of municipalities; therefore, the promotion of ecological civilization in these areas should be carried out in accordance with natural zoning in order to implement multi-sectoral and cross-regional collaborative governance.

### 4.4. Limitations and Prospects

Although the content of the Regulations on the Protection and Utilization of Blackland in Heilongjiang Province regarding LULC control was used as a constraint in predicting future land use changes, the impact of specific measures on land-scape patterns, such as erosion control and fertile cultivation layer construction, in the process of black land protection and utilization was ignored due to the limitations of the prediction model and LULC resolution. The study obtained carbon density by referring to the average value of carbon density data for each category, which limits the accuracy of the study. In the future, long-term surveys and extensive field measurements are required to obtain more detailed data and to account for the heterogeneity of carbon density using a variety of remote sensing techniques [81]. The landscape pattern is influenced by scale effects, and the study divided the evaluation grid of CS and LER only by referencing previous studies, even though the correlations met the study’s criteria. However, there was no comparison to test the best scale revealed by the correlation between variables, and future research should attempt to eliminate the influence of scale effects [82,83]. In addition, the study of green space alone is insufficient to reveal the correlation effects between ecosystem services and landscape ecological risks, and it will be crucial for future research to improve the emphasis on ecosystem wholeness from a multidimensional perspective when examining their interactions.

## 5. Conclusions

Using Heilongjiang province as the study area, we applied the CA–Markov model, InVEST model, landscape ecological risk model, coupled coordination degree model, and spatial autocorrelation analysis to assess the potential impact of BCU policy implementation until 2030 on the relationship between green space CS and LER. The primary findings are as listed below.

(1) From 2020 to 2030, the area of green space in HLJP will exhibit a decreasing trend; however, the BCU measure will minimize the loss of 8492 km^2^ of green area.
(2) In 2030, compared to the NP scenario, the BCU scenario will eliminate the 107.44 × 10^6^ t CS loss caused by the evolution of the green area. The BCU scenario can successfully reduce the total LER level in green space. The green space exchange condition influences, to some extent, the spatial pattern of CS change and LER change.
(3) In 2030, under the BCU scenario, the interdependence between CS and LER is greater than under the NP scenario, but the degree of coupling coordination is slightly lower, and mainly reduces the coupling coordination in the southeast of HLJP.
(4) From 2020 to 2030, there is a significant negative correlation between changes in CS and LER in the green space expansion state, while the two variables are positively correlated in the exchange and loss states. The BCU scenario will considerably increase the size of the H–L sectors, and Heihe City and Jiamusi City are ideal regions for future green spatial evolution for carbon sequestration advantages and risk management.

Overall, this study evaluates the effect of land use policies on the relationship between carbon sequestration and land ecological risk, and can provide a scientific basis for achieving carbon neutrality goals and preventing ecological risk. Using green space as a vehicle for the study has the potential to serve as a connection for cross-sectoral decision-making, and can contribute to the improvement of the interface between regional land use policies and green space system planning. From the standpoint of the evolutionary process of landscape patterns, this study examines the link between CS and LER alterations. Compared to earlier studies, this study demonstrates more correlation effects between ecosystem services and landscape ecological risks. Future studies should focus on uncovering both fundamental mechanisms from a multidimensional perspective.

## Figures and Tables

**Table 1:** Carbon intensity of different land use types (t/ha).

LULC	*C_above*	*C_below*	*C_soil*	*C_dead*
Cultivated land	10.1	26.8	147	0
Forest	11.46	31.32	173.9	2.02
Grassland	7.96	51	74.6	2.84
Water	8.72	2.21	23.01	0
Construction land	8.75	4.39	27.78	1.16
Unutilized land	10.03	0	44.79	0

**Table 2:** The formula for calculating the landscape index and its ecological significance.

Landscape Pattern Index	Calculation Formula	Ecological Meaning
Landscape fragmentation index *C_i_*	$C_{i}=n_{i}∕A_{i}$	Indicates the degree of spatial fragmentation of the landscape, with a higher value indicating a greater human disturbance of the landscape pattern [48].
Landscape separation index *S_i_*	$S_{i}=\frac{A}{2A_{i}}\sqrt{\frac{n_{i}}{A}}$	Indicates the degree of separation between the individual distributions of different patches in a certain landscape type, with greater values indicating a more complex landscape distribution [49].
Landscape dominance index *K_i_*	$K_{i}=\frac{1}{4}\left(\frac{n_{i}}{N}+\frac{m_{i}}{M}\right)+\frac{A_{i}}{2A}$	This shows the significance of landscape patches, with greater values indicating a more homogeneous landscape type [50].
Landscape disturbance index *I_i_*	$I_{i}=aC_{i}+bS_{i}+cK_{i}$	Indicating the degree of loss of several landscape types following a disturbance [51].
Landscape vulnerability index *E_i_*	Drawing on relevant studies in similar natural environment areas to obtain	Larger numbers indicate more susceptibility to external environmental disturbances [52].
Landscape loss index *R_i_*	$R_{i}=U_{i}\times E_{i}$	Indicating the degree of ecological damage caused by various landscape types as a response to external disturbances [53].
Landscape vulnerability index *ERI*	$ERI=\overset{n}{\underset{i=1}{\sum }}\frac{A_{i}}{A_{n}}R_{i}$	Describe the degree of combined ecological loss across many landscape types, with larger values indicating greater ecological risk [54].

Note: $n_{i}$ is the number of patches of landscape i, $A_{i}$ is the area of landscape type i, and A is the total area of ecological risk plots. $m_{i}$ is the number of samples in which patches of landscape type i occur, M is the total number of samples, N is the total number of patches, n is the number of landscape types, and $A_{n}$ is the area of the nth risk plot. Referring to the basis of previous studies [56], *a*, *b* and *c* respond to the weights of each index landscape with 0.5, 0.3 and 0.2, respectively, and the vulnerability indices *Ri* for built-up land, watershed, grassland, forest land, unused land and cropland are normalized to 0.048, 0.095, 0.143, 0.191, 0.238 and 0.286, respectively.

**Table 3:** Evolutionary area of green space (GS) in 2020–2030.

Study Period	GS Expansion	GS Exchanged	GS Loss
	Area (km^2^)	Area (km^2^)	Area (km^2^)
2020–2030 NP	13,422	18,186	30,244
2020–2030 BCU	19,549	41,130	27,879

**Table 4:** The coupling degree of coordination between carbon storage and landscape ecological risk in green space.

Study Period	CS	LER	C	T	D
2020	0.690	0.813	0.997	0.751	0.865
2030 NP	0.651	0.759	0.997	0.705	0.838
2030 BCU	0.666	0.713	0.999	0.689	0.830

## Data Availability

Not applicable.
